# Inoculation to Prevent Yellow Fever

**Published:** 1885-11-20

**Authors:** 


INOCULATION TO PREVENT YELLOW FEVER.—We note that a correspondence is going on between Prof. J. McF. Gaston, of the Southern Medical College of Atlanta, and Dr. Holt, president of the State Board of Health, at New Orleans, relative to "the wonderful discovery of a Brazilian physician," in which he claims that yellow fever can be prevented by inoculation. Professor Gaston is impressed with the truth, and great importance, of the discovery, especially to our Southern section, and desires that the American Public Health Association will take hold of the matter. It is gratifying to know that Professor Gaston has secured the co-operation of so prominent a man as Dr. Holt; and, when we consider the ability and persistence of Professor Gaston in any matter which he undertakes, we feel quite assured that prompt action will be taken, and perhaps important results will be accomplished in this matter.

